# Migraine and Stroke: What’s the Link? What to Do?

**DOI:** 10.1007/s11910-017-0729-y

**Published:** 2017-03-10

**Authors:** Anna Gryglas, Robert Smigiel

**Affiliations:** 1 Department of Neurology, Gromkovski Voivodship Hospital, Wroclaw, Poland; 2 Department of Pediatric Propedeutics and Rare Diseases, Wroclaw Medical University, Wroclaw, Poland

**Keywords:** Migraine, Migraine with aura, Ischemic stroke, White matter lesions, Migrainous infarct, Migraine-related stroke

## Abstract

Migraine and stroke are common, disabling neurologic disorders, with a high socioeconomic burden. A link between them has been proposed years ago, and various theories have been proposed to explain this bidirectional relation. However, the precise causes remain unclear. We briefly summarize existing hypotheses of this correlation seeking for recommendations for stroke prevention in migraineurs, if any exist. Among the strongest suggested theories of migraine–stroke association are cortical spreading depression, endovascular dysfunction, vasoconstriction, neurogenic inflammation, hypercoagulability, increased prevalence of vascular risk factors, shared genetic defects, cervical artery dissection, and patent foramen ovale. There is no evidence that any preventive therapy in migraineurs should be used to decrease stroke risk, even in most predisposed subset of patients. However, a woman with migraine with aura should be encouraged to cease smoking and avoid taking oral contraceptives with high estrogen doses. We need further investigation to better understand the complexity of migraine–stroke association and to make firm recommendations for the future.

## Introduction

A complex association between migraine and stroke has been discussed for over 40 years [1]. These 2 diseases, one seemingly benign and the second often severe and catastrophic, remain common neurologic disorders with a high socioeconomic burden [2•]. Stroke is the second most common cause of death worldwide and the third most common cause of disability [3, 4]. The prevalence of all strokes in adults has been estimated at 2.9% with unclear predominance among men [5, 6]. Migraine affects about 13% of the population and the prevalence of the disease is highest in women in reproductive ages [7, 8]. Numerous epidemiologic studies report that an overall number of stroke patients, stroke related death and global burden of stroke have an increasing tendency [2•]. It is also observed in a population of migraineurs [9]. Emerging evidence indicates that migraine is associated with white matter lesions, cardiac events and vascular diseases as well [10–12]. Among cardiovascular diseases occurring more frequently in migraineurs are hypertension, Reynaud’s syndrome, ischemic heart disease and myocardial infarction, comorbidities which often accompany stroke [13–15]. A recent large prospective cohort study revealed that migraine increases the risk of myocardial infarction (hazard ratio [HR] 1.39), stroke (HR 1.62), angina/coronary revascularization procedures (HR 1.73), and cardiovascular mortality (HR 1.37) [13]. There is some evidence supporting the clinical association between migraine and hemorrhagic stroke and between migraine and transient ischemic attacks [16, 17], but a discrepancy in the literature over the strength and significance of this relationship still remains, as other studies show no association [18]. Undoubtedly the association between migraine and ischemic stroke is the strongest. The percentage of cryptogenic ischemic stroke in migraineurs tends to be higher than in controls.

## Migraine-Related Stroke, Migraine Infarction, and White Matter Hyperintensities

Up to a third of migraineurs experience transient neurologic symptoms called aura, before or during headache [19]. The risk of stroke is twice as high in migraine with aura patients, both between (migraine related stroke) and during attacks (migraine infarction) [20–22]. On the other hand, most studies revealed that migraine without aura does not increase the risk of stroke [11, 23]. Undoubtedly there is a correlation between the frequency of migraine attacks and the risk of stroke [11, 24]. Stroke Prevention in Young Women study revealed a higher stroke risk among women having more than 12 attacks per year [25]. It has also been demonstrated that the stroke risk among migraineurs is elevated in women, among smokers, in women taking oral contraceptive pills, with recent onset of migraine, and in patients younger than 45 [11]. Interestingly, in a population aged 65+ non-migrainous headache, rather than migraine, may be the risk factor for stroke [26]. Migraine infarction is a complication of an attack of migraine with aura. It is an uncommon condition. It occurs with the prevalence 1.4–3.4/100 000, which is 0.2%–0.5% of all ischemic strokes [27, 28]. According to the International Classification of Headache Disorders (ICHD)-3, migrainous infarction is defined as an attack of migraine with aura in which one or more aura symptoms last more than 60 minutes and neuroimaging demonstrates an ischemic brain lesion in the relevant territory. It mostly affects young women with a history of migraine with aura [29]. Studies show 65%–82% of migraine infarction cases are located in the posterior circulation, mostly in the cerebellum [27–31]. There are data revealing that the cerebellum is more vulnerable in migraineurs, particularly with aura, and in familial hemiplegic migraine [32]. Acute ischemic lesions are usually multiple, round- or oval-shaped, with a mean diameter of 7 mm, and located in different arterial areas [27, 28, 31]. Magnetic resonance imaging of migraine brain, particularly T2 –weighted and FLAIR sequences, often reveal small, usually multiple hyperintensities, typically located periventricularly or in the deep white matter, which are called white matter lesions (WML) [33, 34]. WML are not specific to migraine; however, their prevalence ranges from 4% to 59% of migraine population, which is significantly higher than in controls [35]. The exact etiology and clinical significance of these abnormalities remains uncertain. Some data show that the intensification of these abnormalities increases with migraine frequency (in those with ≥1 attack/month OR = 2.6; 95% CI 1.2–6.0) (26 Kruit 2010). A study evaluating if cognitive decline could be related to WML failed to find any association; however, there are reports indicating an enhanced risk of stroke with increase in WML volume [36]. Further investigation is needed, considering that fact that we can speculate that brain structural changes in migraine could serve as disease biomarkers and help to evaluate the risk of stroke in the future.

## The Underlying Mechanisms of Coincidence Migraine and Stroke

The pathogenic mechanisms explaining the migraine–stroke association remain poorly understood, but a few hypotheses exist. Among suggested theories of migraine–stroke relation are cortical spreading depression, vasoconstriction, endovascular dysfunction, increased prevalence of vascular risk factors, shared genetic defects, cervical artery dissection, cardiac abnormalities including patent foramen ovale (PFO), neurogenic inflammation, and hypercoagulability [11, 19, 37–43].

### Cortical Spreading Depression

Cortical spreading depression (CSD) is the electrophysiological mechanism that underlies migraine aura [44]. Also, it plays a role in the pathogenesis of ischemic stroke, intracranial hemorrhage, subarachnoid hemorrhage, traumatic brain injury, transient global amnesia, and some other diseases [45–47]. CSD is a potent, self- propagating, short-lasting depolarization wave that moves across the cortex at a rate of 3–5 mm/min [48]. By activation of metalloproteinases, CSD changes the permeability of the blood–brain barrier [49]. Therefore, it is associated with significant ionic and water changes and large transient increase in energy metabolism [50]. Higher oxygen and glucose expenditure accompanying CSD raises cerebral blood flow [47, 51]. Cerebral hyperemia is followed by prolonged cerebral oligaemia attributable to decreased vascular responsiveness [52]. Therefore, it may favor ischemic events in the brain [53]. In addition, in vulnerable tissue, severe hypoperfusion may follow CSD, consequently leading to enlargement of ischemic lesion [54]. Mice with human migraine mutations represent an increased vulnerability to CSD and cerebral ischemia [37, 53]. It may be speculated that CSD could be the mechanism causing the ischemic lesion in the vulnerable brain.

### The Role of Endothelium

The role of endothelium in migraine pathogenesis and migraine-associated stroke has been raised during the past decades [55]. Endothelium participates in homeostasis influencing a vascular reactivity, but also thrombolysis, thrombosis, proliferation, and apoptosis [56]. Microparticles released from endothelial cells lead to endothelial activation and dysfunction [57]. Among endothelial biomarkers of injured endothelium are vascular endothelial growth factor, t- PA, von Willebrand factor, C-reactive protein, reduced nitrate levels, all of which were found in migraineurs, some only in migraineurs with aura [58, 59]. Inflammatory cytokines released in the aura phase activate the endothelium leading to procoagulatory and prothrombotic state [55]. However, research of cerebrovascular reactivity in migraineurs has demonstrated conflicting data (decreased or increased cerebral vascular reactivity) in regard to the causal relationship of endothelium dysfunction in stroke pathogenesis [60–62].

### Cardiovascular RF

Migraineurs are at the heightened risk of various types of vascular events including stroke [63]. For this reason, stroke risk factors in migraineurs have been widely evaluated in the literature. Regarding hypertension, which may impair cerebral blood flow and cause alterations in cerebral artery structure, we find studies indicating the incidence of high blood pressure is higher in migraine patient and contradictory evidence from other research [64–68]. Similarly, the data about the relationship of migraine and obesity are inconsistent. Most studies show that obesity increases the risk of having migraine and the risk grows with increasing BMI [69, 70]. Total body obesity may impact migraine frequency, severity, and duration [71]. Subsequently, a large body of evidence has highlighted an involvement of obesity in migraine chronification [70–72]. The majority of available studies indicate that there is a positive correlation between cigarette smoking and migraine, revealing that the number of smokers among migraineurs is significantly higher compared with healthy controls [72–74]. Smoking among migraineurs seems to be related to the development of cranial autonomic symptoms, but evidence indicating that tobacco use may trigger headache is lacking [75, 76]. However, Coronary Artery Risk Development in Young Adults (CARDIA) Study demonstrated an increased risk of developing migraine in young smokers [77]. Studies on the role of dyslipidemia in migraine patients are quite homogenous and demonstrate an association between unfavorable cholesterol profile and migraine, particularly with aura [14]. Fewer studies failed to demonstrate the association between dyslipidemia and migraine or showed negative association [72, 78]. Based on the available data, we cannot indicate any single risk factor that could play a predominant role in the pathogenesis of stroke in migraineurs.

### Patent Foramen Ovale

Patent foramen ovale (PFO) is linked to cryptogenic stroke and transient ischemic attacks in a mechanism of paradoxical embolism and transient hypoxemia [79–81]. Many, but not all available studies report there is a higher prevalence of PFO in migraineurs, especially with aura [82–86]. Wolff reported 64.7% of migrainous infarction patients have persistent foramen ovale [28]. As demonstrated by Larossa, PFO is not more common or larger in chronic migraine than in episodic migraine patients [85]. In a subset of migraineurs with stroke, prevalence of PFO was significantly higher and the mean age of stroke was lower than in controls [87]. Numerous observational studies report positive impact of PFO closure on the occurrence of migraine [88–91]. However, the balance between risks and benefits is not favorable enough; therefore, the routine detection and closure of PFO for migraine prevention is not recommended [84, 92, 93].

### Underlying Genetic Disorders

The complexity of the migraine and stroke correlation is seen in genetic vasculopathies such as cerebral autosomal dominant arteriopathy with subcortical infarcts and leukoencephalopathy (CADASIL). Mitochondrial encephalopathy with lactic acidosis and stroke (MELAS), cerebroretinal vasculopathy, and hereditary endotheliopathy with retinopathy, nephropathy and stroke (HERNS) are other genetic disorders connecting migraine and stroke-like episodes; however, these interesting topics remain beyond the scope of this article. Apart from these disorders, other genetic factors seem to play a role in the discussed correlation. Data from a recent, large, genome-wide analysis study indicate that the strongest genetic overlap exists between migraine without aura and large artery stroke, and cardio-embolic stroke [94]. Genetic factors do not seem to play an important role in a shared pathogenesis of stroke and migraine with aura [94].

### Cerebral Autosomal Dominant Arteriopathy with Subcortical Infarcts and Leukoencephalopathy

Cerebral autosomal dominant arteriopathy with subcortical infarcts and leukoencephalopathy (CADASI) is an autosomal dominant disease caused by rare mutations of the gene encoding the neurogenic locus notch homolog protein 3 (NOTCH 3 gene) located on chromosome 19p13 [95]. The gene encodes a vascular smooth muscle cells receptor. Its defect leads to chronic white matter ischemia due to non-artheriosclerotic, non-amyloid vasculopathy with fibrosis and an accumulation of granular and osmiophilic substances perforating cerebral arteries [96, 97]. Stroke risk among these patients is enhanced. It should be suspected when a patient has one or more of recurrent subcortical ischemic strokes at the mean age of 45 years, especially in the absence of other risk factors, migraine (typically with aura) and/or early cognitive decline, or subcortical dementia usually associated with pseudobulbar palsy [34, 98]. Migraine, typically with aura, occurs in about 30% of patients. It is usually the earliest symptom, and in some patients the only clinical manifestation of the disease [98]. In most cases attacks of migraine improve or cease when other manifestations of CADASIL (e.g., ischemic stroke) appear [29]. Magnetic resonance imaging is always abnormal with well delineated, small, bilateral infarcts typically seen in the deep white matter and in the periventricular region predominantly in the frontal, parietal and anterior temporal lobes, rarely in the occipital lobes [99].

Familial hemiplegic migraine (FMH) is a rare (5/100 000), autosomal dominant disease, the subtypes of which are caused by mutation on the CACNA1A, ATP1A2, SCN1A or PRRT2 genes [34, 100, 101]. FHM is characterized by reversible motor weakness, which may resemble stroke or transient ischemic attack [29]. Studies indicate that FHM increases susceptibility to spreading depression and enhances ischemic vulnerability of migraine brain [102].

### Migraine Drugs

Migraine specific drugs, triptans and ergotamines, may induce vasoconstriction that putatively could enhance stroke risk [103]. Several studies on triptan safety reveal conflicting results, so no firm conclusion can be drawn [104, 105]. However, by analyzing the available data we may speculate that even if the absolute risk of stroke is enhanced in patients recently exposed to triptans, the risk is small and estimated at 1:100,000 treated attacks for sumatriptan [106]. Therefore, in light of current knowledge, minding their effectiveness in treating migraine we should always take triptans into consideration as a treatment option. As migraine is a disorder that particularly concerns young woman in the fertile period of life, contraceptive pills are often required [107]. Furthermore, in menstrual related migraine, stabilizing estrogen level by use of contraceptive pills may decrease the intensity and duration of menstrual related migraine attacks [108, 109]. Some headache specialists are still cautious with their use for fear of increasing stroke risk. Most of the past studies evaluating the effect of oral contraceptive pills on the course of migraine and stroke risk were based on high dose estrogen pills 50 to 100 mcg of ethynyl estradiol. Current evidence demonstrates that new, low-dose oral contraceptives (25 mcg of ethynyl estradiol or less) do not enhance vascular risk in migraine woman [110].

## Conclusions

A large body of evidence supports the link between migraine and ischemic stroke. The subset of patients at the most increased risk of stroke are young females with migraine with aura and patients suffering from genetic disorders like CADASIL, MELAS, and HERNS. A 2-fold increased risk of ischemic stroke in migraine with aura patients has been noted in some population-based studies [22, 29, 111]. Migraineurs, particularly at heightened risk of stroke, should be encouraged to manage other risk factors for stroke, like hypertension, diabetes, obesity, elevated homocysteine, and dyslipidemia, as it seems to be a reasonable approach to decrease the overall risk of stroke. A firm recommendation not to smoke should be made. Evidence suggests that we should not be afraid of using low-estrogen contraceptives in females with migraine, especially when non-hormonal preventive treatment has been unsuccessful [107, 112]. A firm correlation between the frequency of migraine attacks and the risk of stroke exists; however, there are no data indicating that migraine prophylaxis decreases the risk of migrainous infarct [113]. Keeping in mind the aspect of quality of life in migraineurs, we should always consider use of migraine-specific abortive drugs, remembering that in female migraineurs the absolute risk of stroke is low [114]. Considering that we lack sufficient proof to justify any of the known factors having a predominant role in migraine stroke relation, further investigation is a necessary. It should focus on answering the question whether stroke in migraine with aura patients might be prevented, and by which therapeutic strategies.
